# Low NETosis Induced in *Anaplasma phagocytophilum*-Infected Cells

**DOI:** 10.3390/vaccines10101756

**Published:** 2022-10-20

**Authors:** Sara Artigas-Jerónimo, Almudena González-García, José de la Fuente, Valeria Blanda, Mojtaba Shekarkar Azgomi, Margarita Villar, Leila Mohammadnezhad, Francesca Grippi, Alessandra Torina, Guido Sireci

**Affiliations:** 1 SaBio. Instituto de Investigación en Recursos Cinegéticos IREC-CSIC-UCLM-JCCM, Ronda de Toledo s/n, 13005 Ciudad Real, Spain; 2 Department of Veterinary Pathobiology, Center for Veterinary Health Sciences, Oklahoma State University, Stillwater, OK 74078, USA; 3 Istituto Zooprofilattico Sperimentale Della Sicilia, Via Gino Marinuzzi 3, 90129 Palermo, Italy; 4 Central Laboratory of Advanced Diagnosis and Biomedical Research (CLADIBIOR), University of Palermo, 90129 Palermo, Italy; 5 Department of Health Promotion, Mother and Child Care, Internal Medicine and Medical Specialties, University of Palermo, Via del Vespro 129, 90127 Palermo, Italy; 6 Biochemistry Section, Regional Centre for Biomedical Research (CRIB), Faculty of Science and Chemical Technologies, University of Castilla-La Mancha, 45071 Toledo, Spain; 7 Department of Biomedicine, Neurosciences and Advanced Diagnostic (Bi.N.D.), University of Palermo, Via del Vespro 129, 90127 Palermo, Italy

**Keywords:** neutrophils, NETosis, *Anaplasma phagocytophilum*, ROS

## Abstract

*Anaplasma phagocytophilum* are obligatory intracellular bacteria that preferentially replicate inside leukocytes by utilizing biological compounds and processes of these primary host defensive cells. In this study, bioinformatics analysis was conducted to further characterize *A. phagocytophilum*–host interactions using the neutrophil-like model of human Caucasian promyelocytic leukemia HL60 cells. We detected a hierarchy of molecules involved in *A. phagocytophilum*-HL60 interactions with overrepresentation in infected human cells of proteins involved in the reactive oxygen species (ROS) pathway and cell surface monocyte markers. As *A. phagocytophilum* phagocytosis by neutrophils is inhibited, the results suggested a possible explanation for our bioinformatics data: radical oxygen compounds could induce the killing of bacteria activating NETosis, a unique form of defense mechanism resulting in cell death that is characterized by the release of decondensed chromatin and granular contents to the extracellular space, forming neutrophil extracellular traps (NETs) to eliminate invading microorganisms. Thus, we confirmed the existence of a low NETosis induced in *A. phagocytophilum*-infected cells by immunofluorescence (IF) experiments. These results provide new insights into the complex mechanisms that govern immune response during *A. phagocytophilum* host interactions.

## 1. Introduction

*Anaplasma phagocytohilum* is an intracellular bacterium responsible for tick-borne diseases that infect humans and animals worldwide [1]. In humans, *A. phagocytophilum* causes human granulocytic anaplasmosis (HGA), infecting host granulocytes, such as neutrophils, and previous publications show how these cells are able to control *A. phagocytophilum* infection [2]. 

Upon interaction with host monocytes and granulocytes, *A. phagocytophilum* usurps the lipid raft domain-containing glycosylphosphatidylinositol (GPI)-anchored proteins, integral membrane proteins present in the cell surface that can perform multiple functions [3], inducing a series of signaling events that result in the cell internalization of the bacteria. *A. phagocytophilum* alters vesicular traffic to create a unique intracellular membrane-bound compartment that allows their replication in seclusion from lysosomal killing. Moreover, host phagocyte activation and differentiation, apoptosis, and interferon (IFN)-gamma signaling pathways are inhibited by these bacteria [4]. In the trade-off against the bacteria, monocytes and neutrophils usually kill invading microorganisms by fusion of the phagosomes containing the bacteria with granules containing both antimicrobial peptides and lysosomal hydrolytic enzymes and/or through sequestering vital nutrients [5]. Neutrophils play a key role in the immune protection system as innate immune phagocytes [6,7,8]. One of the key mechanisms induced by neutrophils is the release of neutrophil extracellular traps (NETs), which are web-like structures of chromatin together with molecules such as histones and myeloperoxidase (MPO) with a protective role in the host’s immune defense [6,7,8,9,10]. The process of NETs release is known as NETosis with slow cell death and non-lytic rapid release from live cell pathways [8]. Undifferentiated human promyelocytic cells (HL-60) may be differentiated into neutrophil-like cells using dimethyl sulfoxide (DMSO). Under experimental conditions, differentiated HL60 cells are efficient at producing NETs upon phorbol myristate acetate (PMA) stimulation in a serum-free medium [8,9]. The components that integrate NETs may vary depending on the pathogen which the neutrophil is facing [8]. NETs are also composed of neutrophil elastase (ELANE) which together with MPO form the MPO-ELANE pathway with a role in NETosis and NETs formation induced by stimuli such as bacteria, fungi, and crystals [8]. 

As bacteria, *A. phagocytophilum* activates the innate immune response in human neutrophils, but there is no previous report of NETosis in response to this bacterium. So, the main goal of this study is the evaluation of *A. phagocytophilum* as the trigger of the neutrophil NETosis as well as the identification of the NETosis signal pathway during infection with *A. phagocytophilum.*

## 2. Materials and Methods

### 2.1. Bioinformatic Analysis

From previously published proteomics data [11], 21 human genes (Dataset S1) were chosen based on a cut-off *p*-value < 0.05. Those 21 human genes were inserted into Cytoscape (3.9.1) to predict a Molecule–Protein interaction. Then, we used Gene Ontology (GO), and visualization, interpretation, and analysis of the reactome pathway was run by G: profiler analysis [12] to find the functions of related genes and the involved biological pathways, respectively. Finally, we hypothesized the existence of the NETosis pathway using the STITCH database, a tool for predicting chemical–protein and protein–protein interactions based on computational prediction [13].

### 2.2. HL60 Cell Culture

HL60 undifferentiated promyelocytic leukemia cells, a validated cellular model for the study of different blood cell types as human neutrophils [14], were cultured in 25 cm^2^ flasks containing RPMI 1640 medium (Gibco, ThermoFisher Scientific, Waltham, MA, USA) supplemented with 10% heat-inactivated fetal calf serum (Gibco, ThermoFisher Scientific) at 37 °C with 5% CO_2_, as previously described [15]. 

### 2.3. Anaplasma phagocytophilum-Infected HL60 Cells and Bacteria Isolation

The *A. phagocytophilum* human NY18 isolate, a well-known strain that affect humans worldwide [11,16,17], was propagated in cultured HL60 cells as previously described [15]. The *A. phagocytophilum*-infected cells were collected when 70–80% of the cells were infected as determined by the detection of intracellular morulae in stained cytospin cell smears. Cell-free bacteria were isolated from cell culture by passing the cells 5–10 times through a 21-gauge syringe, then centrifuged at 300× *g* for 5 min and resuspended in X-VIVO 15 media (Lonza, Cultek S.L., Madrid, Spain).

### 2.4. HL60 Differentiation and NETs Stimulation

HL60 cells differentiated into neutrophils after adding 1.25% DMSO for 5 days. DMSO was added every 2 days to the cells. Then, differentiated HL60 cells (1 × 10^6^ cells/well) were seeded in a 12-well plate in X-vivo serum-free medium (Cultek S.L.). After 1 h incubation at 37 °C with 5% CO_2_, cells were stimulated with 50 nmol/L phorbol myristate acetate (PMA) (ab120297; Abcam, Cambridge, UK) as the positive control. *Escherichia coli* 086 or *A. phagocytophilum* free cells with a OD_600_ = 0.9–1.4 were inoculated to the cultured cells. X-vivo medium were used as the negative control. Cells were then incubated for 4 h and harvested for flow cytometry and immunofluorescence analyses. *E. coli* O86, a pathogenic strain, was grown in Lysogeny broth (LB) medium (VWR, Radnor, PA, USA) overnight at 37 °C with shaking before the experiment. 

### 2.5. Annexin V-FITC Staining to Detect Cell Apoptosis

Approximately 5 × 10^5^ cells were collected from the experimental plates immediately after treatment incubation for 4 h. All treatments were conducted in duplicate. Apoptosis was measured by flow cytometry using the Annexin V-fluorescein isothiocyanate (V-FITC) apoptosis detection kit (Immunostep S.L., Salamanca, Spain) following the manufacturer’s protocol. The kit allows the discrimination of intact cells (Annexin V-FITC negative, propidium iodide (PI) negative), and early apoptotic cells (Annexin V-FITC positive, PI negative). All samples were analyzed on a FACScalibur flow cytometer equipped with CellQuest Pro software (BD Biosciences, Madrid, Spain) and FlowJo v10 software (BD Biosciences). The percentage of apoptotic cells was determined by flow cytometry after Annexin V-FITC and PI labeling and compared between treatments by Student’s *t*-test with unequal variance (*p* < 0.05).

### 2.6. Live/Dead Cells and MPO Quantification by Flow Cytometry

To evaluate MPO intracellular levels, 1 × 10^6^ cells per replicate (4 replicates/treatment) were collected and washed with 2 mL of 10 mM PBS, 0.5% BSA, 0.065% sodium azide. Cell viability was measured using a Live/Dead Fixable Green Dead cell stain Kit (Molecular Probes, Eugene, OR, USA) following the manufacturer’s protocols. Cells were next fixed and permeabilized using the cell fixation and permeabilization kit (Immunostep). Five µL of human BD Fc Block (BD Biosciences) were added to block or significantly reduce potentially non-specific antibody staining caused by receptors for IgG in each sample. Then, 1:50 anti-MPO antibodies (ab9535, Abcam) were added, and samples were washed twice with 10 mM PBS, 0.5% BSA, and 0.065% sodium azide. Finally, fluorescently labeled Alexa Fluor 635 goat anti-rabbit IgG secondary antibody (A31576; Invitrogen, Carlsbad, CA, USA) was added at a concentration of 10 µg/mL. MPO fluorescence intensity was measured through flow cytometry FACS Calibur (BD Biosciences) and analyzed using FLowJo v10 software (BD Biosciences). The MPO geometrical mean fluorescence intensity was measured in the viable cell population gated according to green live/dead cell stain. The MPO geometrical mean fluorescence intensity was compared between treatments by one-way ANOVA with post hoc Tukey HSD (https://astatsa.com/OneWay_Anova_with_TukeyHSD/; accessed on 28 April 2022) (*p* < 0.05; *n* = 4 biological replicates).

### 2.7. Assessment of HL60 Differentiation by Flow Cytometry

Approximately 10^6^ HL60 cells per treatment were incubated with 5 µL of mouse Phycoerythrin (PE) anti-human CD16 antibody (#360703; BioLegend, San Diego, CA, USA) for 30 min at RT in the dark, and then, cells were washed with 1 mL 10 mM PBS + 0.5% BSA+ 0.065% sodium azide. All samples were analyzed on a FACScalibur flow cytometer equipped with CellQuest Pro software (BD Biosciences) and FlowJo v10 software (BD Biosciences). The CD16 binding-capacity and thus cell differentiation were measured in comparison with DMSO-induced HL60 cells without incubation with CD16 antibody (Figure S1). 

### 2.8. Immunofluorescence Assay (IFA) of HL60 Cells

Cells were fixed with 4% paraformaldehyde at room temperature (RT) for 30 min and then, permeabilized with 0.3% Triton X-100 (Sigma-Aldrich, St. Louis, MO, USA) for 30 min at RT and 0.1% SDS for 10 min at RT. Then, slides were washed three times with PBS 10 mM for 5 min and blocked with 10% goat serum (G9023; Sigma-Aldrich) for 1 h at RT. Slides were rinsed with PBS to wash cells. Then, cells were incubated overnight at 4 °C with 1:100 anti-MPO primary antibody (ab9535; Abcam). After three washes with PBS of 5 min each, cells were incubated with a fluorescently labeled Alexa Fluor 635 goat anti-rabbit IgG secondary antibody (A31576; Invitrogen) for 1.5 h at RT in darkness. Then, slides were washed four times with PBS for 5 min each. Cells were counterstained with ProLong Antifade containing 4′,6-diamidino-2-phenylindole (DAPI) (Molecular Probes, Eugene, OR, USA; blue-stained) and imaged with a Zeiss LSM800 confocal microscope using 63× oil immersion lens (Carl Zeiss, Oberkochen, Germany). 

### 2.9. Determination of A. phagocytophilum DNA Levels by qRT-PCR

Total genomic DNA was extracted from *A. phagocytophilum*-infected and uninfected HL60 cells using DNeasy Blood and Tissue Kits (Qiagen, Hilden, Germany) following the manufacturer’s recommendations. The *A. phagocytophilum* DNA levels were characterized by real-time PCR using gene-specific oligonucleotide forward (F) and reverse (R) primers for major surface protein 2 (msp2) gene (F: 5′-ATGGAAGGTAGTGTTGGTTATGGTATT-3′ and R: 5′-TTGGTCTTGAAGCGCTCGTA-3′) and Luna Universal qPCR Master Mix (New England Biolabs Inc., Ipswich, MA, USA). A dissociation curve was run at the end of the reaction to ensure that only one amplicon was formed, and the amplicons denatured consistently at the same temperature range for every sample. The cDNA levels were normalized against human protein importin 8 (IPO8; F: 5′-GGCATACAGTTTAACCTGCCAC -3′ and R: 5′-CAGGAGAGGCATCATGTCTGTAA-3′) using the genNorm Delta-Delta-Ct (ddCt) method [18]. Normalized cycle threshold (Ct) values were compared between infected and uninfected HL60 cells by Student’s *t*-test with unequal variance (*p* < 0.05; *n* = 4; Figure S2).

## 3. Results and Discussion

### 3.1. Bioinformatics Analysis Showed Overactivation of NETosis-Related Proteins in Infected Human HL60 Cells

**Figure 1:**
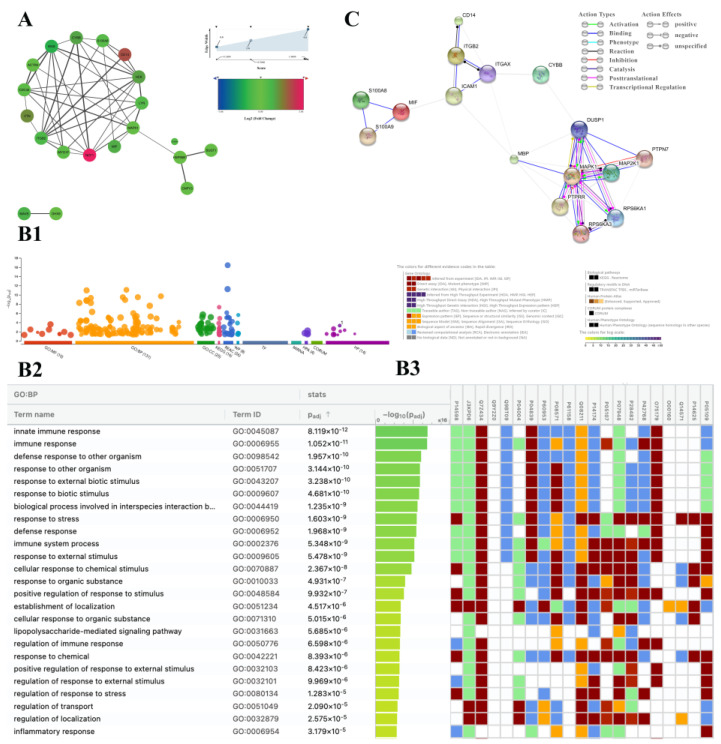
Overactivation of NETosis-related proteins in *A. phagocytophilum*-infected human cells. (**A**) Protein interactions network between proteins that are significantly different in human cell proteomics analysis. The color of each nod is based on Log2 fold change, and edge mapping width is based on protein interaction score: min = 0.2, max = 1.00. (**B1**) Eleven different ontology terms have been used for the go study. (**B2**) GO data shows that proteins are involved in the ‘innate immune system’, ‘response to stress’, and ‘lipopolysaccharide-mediated signaling pathway’ are significant triggers in the network. (**B3**) Reactome data shows the association of the ‘innate immune system’ and ‘neutrophil degranulation’ pathways in our data. (**C**) String network shows a protein-protein interaction implied in NETosis.

The proteomics data obtained from *A. phagocytophilum*-infected human HL60 [11] cells revealed that 21 proteins among 139 proteins had significant fold-changes (*p*-value < 0.05). The most significantly upregulated protein (*p*-value = 0.0005) was neutrophil cytosol factor 1 (NCF1, accession P14598) with log2 fold change = 3.055. The upregulation of monocyte differentiation antigen CD14 (P08571), Cytochrome b-245 heavy chain (CYBB, P04839), and Integrin beta-2 (ITGB2, P05107) was also noteworthy. Moreover, Mitogen-activated protein kinase 1 (MAPK1, P28482) and Protein S100-A8 (S100A8, P05109) were included in the upregulated proteins. Thus, first, it was relevant to find protein-protein interactions (PPI) between these overrepresented proteins. Based on Figure 1A and Dataset S1 we proposed that CD14^+^ cells could be the main subset playing a role in this complex (Log2FC = 2.1, *p*-value = 0.016, Edge Width = 4.0). So, we created a PPI interaction model to discover a network between CD14^+^ cells and the other proteins that were changed significantly in infected cellular subsets. Based on this network (Figure 1A), CD14, ITGB2, CYBB, and NCF1, have higher scores of interactions. Given the role of ITGB2 in the adhesion and transmigration of neutrophils [19,20] and that CYBB and NCF1 genes encode NADPH oxidase 2 (NOX2) subunits [21], we hypothesized that CD14^+^ neutrophils could induce NETosis through NOX2. To achieve this hypothesis and understand gene product attributes, we used gene ontology. In addition, visualization, interpretation of the data using reactome analysis facilitated in identifying the main pathway within this network (Figure 1(B1–B3)). GO analysis has revealed that the proteins related to the ‘innate immune system’ are the most pathway triggers inside the network (P_adj_ 8.119 × 10^−12^), where the existence of the proteins in line with the ‘response to stress’ (P_adj_ 1.603 × 10^−9^), and ‘lipopolysaccharide-mediated signaling pathway’ (P_adj_ 5.685 × 10^−6^) were significantly involved pathways in this network (Figure 1(B2)). Moreover, the reactome database showed the biological pathway associated with ‘neutrophil degranulation’ as a significant biological pathway (P_adj_ 3.738 × 10^−2^) (Figure 1(B3)). These results confirmed our hypothesis that NETosis could be a critical signal in infected cells. So, we used the upregulated proteins associated with neutrophil degranulation (MIF, MAPK1, CD14, S100A8, CYBB, and ITGB2) through the string network to find how the conjunction of all factors plays a role in NETosis. We observed three nodes within the network (Figure 1C). The S100A8 and S100A9 proteins have a role in neutrophil aggregation in the first node (GO:0070488). Aside from those proteins, ITGB2 in the second node is the neutrophils’ chemotaxis factors (GO:0030593). The second node communicates with the third via CYBB, whose gene is required for NOX2 activity and thus in NETosis. Furthermore, MAPK1, MAP2K1, RPS6KA3, and RPS6KA1 in the third node, as well as CD14 and ITGB2, are players in the TLR4 signaling pathway (GO:0034142). Finally, based on these data we concluded that the NETosis signal pathway may be a critical signal during infection with *A. phagocytophilum*.

### 3.2. Anaplasma phagocytophilum and E. coli Differently Affect Differentiated HL60 Cells

**Figure 2:**
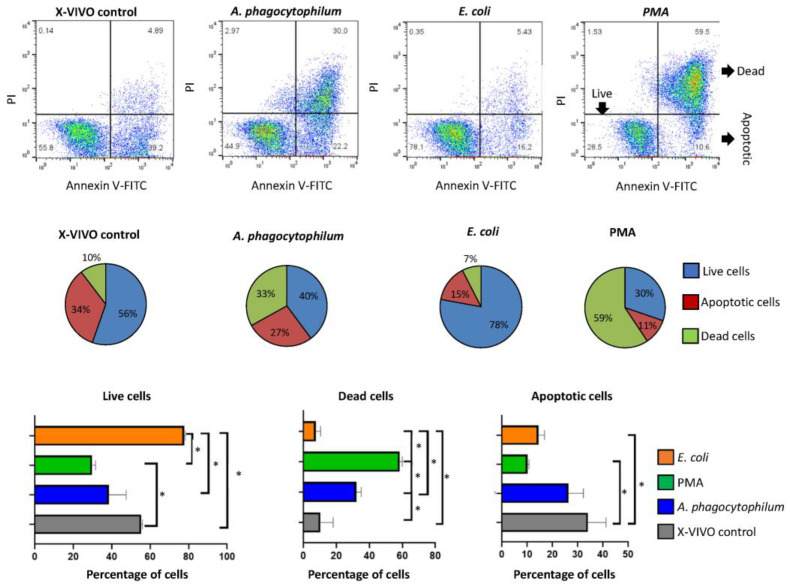
Cellular viability and apoptosis study after different treatments by flow cytometry. Representative flow cytometry analyses where live (Annexin V-FITC negative, PI negative), dead (Annexin V-FITC positive, PI positive), and apoptotic cells (Annexin V-FITC positive, PI negative) are separated regarding their capability of binding PI and Annexin V FITC in the four different treatments support the percentage of live, dead and apoptotic cells after the four treatments, which were compared between treatments by one-way ANOVA test (* *p* < 0.05; *n* = 2 biological replicates).

The evaluation of live, apoptotic, and dead differentiated HL60 cells was carried out after the incubation of cells with four different treatments (X-VIVO control, Ap, *E. coli,* and PMA) (Figure 2). The use of Annexin V-FITC allows us to detect changes in phospholipid symmetry analyzed by measuring Annexin V binding to phosphatidylserine, which is exposed on the external surface of the cell membrane in apoptotic cells. Thus, we can detect intact live, apoptotic, and dead cells by flow cytometry (Figure 2). 

In agreement with previous studies [22], PMA treatment significantly increased the percentage of dead cells when compared with the control and rest of the groups. Treatment with *A. phagocytophilum* significantly increased the percentage of dead cells when compared to control but not to PMA. However, treatment with *E. coli* reduced the percentage of dead cells when compared to all groups (Figure 2). The percentage of apoptotic cells was higher in control treatment when compared to PMA and *E. coli* but not *A. phagocytophilum* treatments (Figure 2). As expected, the incubation with extracellular *E. coli* did not affect differentiated HL60 cells, probably reflecting the inability to infect these cells and its survival within neutrophils after the phagocytosis [23]. These results support previous findings highlighting that *A. phagocytophilum* infection has a low impact on changing human proteome. In particular, such studies showed that human neutrophil cells are able to limit pathogen infection through a differential representation of ras-related proteins [11].

### 3.3. A. phagocytophilum Does Not Increase MPO Expression, and thus, NETs Formation in Differentiated HL60 Cells

**Figure 3:**
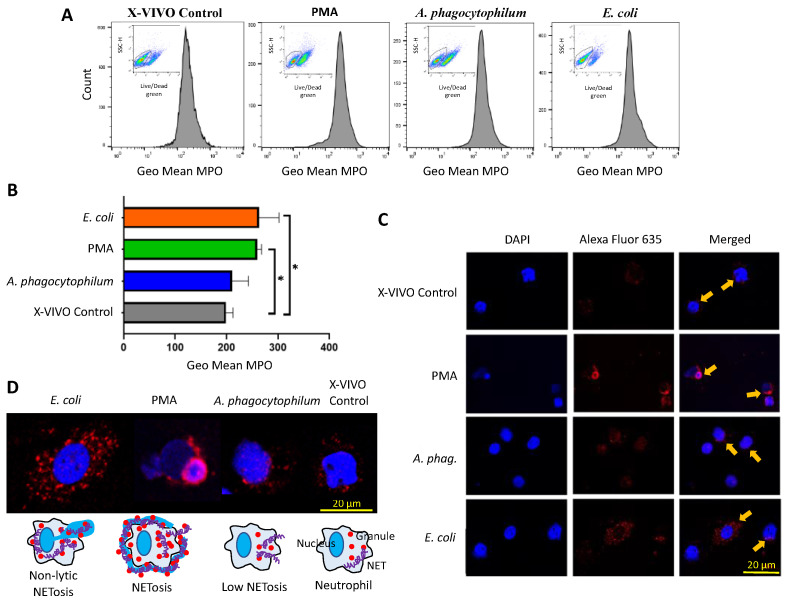
MPO levels in differentiated HL60 cells. (**A**) Live/dead cells were differentiated and quantified in all treatments using a fixable Green Dead cell stain Kit (Molecular Probes, Eugene, OR, USA). Then, live cells were gated to evaluate MPO expression only in these cells. Live cells were detected in the four different treatments. High resolution images are displayed in Supplementary Figure S4. (**B**) The MPO levels were quantified, and the geometric mean (Geo Mean) compared between groups by one-way ANOVA test (* *p* < 0.05; *n* = 4 biological replicates). (**C**) Representative images of immunofluorescence analysis of cells treated with X-VIVO control, PMA, *A. phagocytophilum*, and *E. coli*. Cells were stained with rabbit anti-MPO antibodies and conjugated with fluorescently labeled Alexa Fluor 635 goat anti-rabbit IgG secondary antibody (MPO in red, arrows) and labeled with DAPI (DNA in blue). (**D**) Representative images of immunofluorescence analysis and proposed associated models of induced NETosis.

Flow cytometry analysis was used to identify live and dead cells and to quantify MPO fluorescence only on live cells (Figure 3A and Figure S3). The results corroborated that the viability of differentiated HL60 cells was reduced after PMA treatment in comparison with all groups (Figure 3A and Figure S3). 

The MPO levels were higher in live cells incubated with PMA or *E. coli* in comparison with control cells (Figure 3B). These results showed that differentiated HL60 cells produce NETs upon stimulation with PMA and *E. coli* [8,9]. However, treatment with *A. phagocytophilum* did not significantly alter the MPO levels, supporting that infection with these bacteria do not induce neutrophil NETs formation as a result of co-evolutionary processes to guarantee the survival of both pathogen and host neutrophil cells [11,15,24,25,26].

The immunofluorescence analysis showed differences between treatments in the localization of MPO and associated NETs (Figure 3C,D). In cells incubated with *E. coli*, results showed a high MPO fluorescence in the cytoplasm across the whole cell. In the case of the positive control, PMA, known to induce the formation of NETs, results showed a very compact MPO fluorescence just embracing the neutrophil membrane. In the control sample, MPO was mostly located in the cytoplasm. For *A. phagocytophilum*, MPO was also mainly located in the cytoplasm but also in the nuclear membrane. 

Thus, the fluorescence patterns of MPO in response to different treatments are not equal and suggest differences in host cell NETs-mediated response [8]. While *E. coli* appears to induce a non-lytic NETosis from live cells, PMA produced a NETosis with cell death (Figure 3D). Treatment with *A. phagocytophilum* was associated with low-level NETosis (Figure 3D). 

*Escherichia coli* O86 belongs to the EnteroPathogenic *E. coli* (EPEC) group, where some strains are pathogenic for humans and animals. As an enteropathogenic *E. coli* strain, *E. coli* O86 may trigger an immune response in the host, which may be associated with MPO levels and NETs formation. The *E. coli* O86 O-antigen consists of pentasaccharide O-units with three different sugars: l-fucose (Fuc), d-galactose (Gal), and N-acetyl galactosamine (GalNAc) [27]. Additionally, *E. coli* O86 possesses high human blood group B activity because of its O-antigen structure, sharing the human blood group B epitope [28]. Some *E. coli* O86 serotypes, such as *E. coli* O86:B7, have Galα1-3Galβ1-4GlcNAc-R (α-gal) conjugated to the O-antigen of its LPS. In consequence, *E. coli* O86:B7 is a pathobiont of human microbiota known to likely contribute to the production of anti-α-gal antibodies [29]. The role of human microbiota in health [30] may explain at least in part why *E. coli* treatment showed an increase in differentiated HL60 survival rate.

To investigate the pathways involved in *A. phagocytophilum*-infected neutrophils, we infected human HL60 cells with *A. phagocytophilum* bacteria. Even if *A. phagocytophilum* is surviving in polymorphonuclear granulocytes and affects apoptosis of this cell lineage, deep characterization of possible targets of innate immunity is needed. Thus, bioinformatics analysis was performed on the proteomics data obtained from the in vitro experiment (Dataset S1) in which we found that the main molecules upregulated in *A. phagocytophilum*-infected human HL60 cells (infected/uninfected ratio = 0.456–8.312) are molecules involved in either ROS release or monocyte and neutrophil activation. CD14^+^ is a LPS-binding molecule expressed on monocytes and neutrophils [31], activating these cells to produce proinflammatory cytokines [32]. Moreover, LPS induces neutrophils to the extracellular release of neutrophil extracellular traps (NET) in a NOX-dependent manner [33,34], which is a crucial source of ROS production [35].

Interestingly, our data showed a significant upregulation of NOX2 subunits NCF1 and CYBB in infected cells, but these data contrast with previously published evidence that reported that ROS phagocytosis is inhibited in *A. phagocytophilum*-infected cells. ROS release is not only involved in phagocytosis, but many biological processes like NETosis require ROS secretion [21]. Furthermore, we found an increase in many molecules involved in TLR4 signaling, such as MAPK1, MAP2K1, RPS6KA3, and RPS6KA1, in *A. phagocytophilum*-infected granulocytes by protein–protein interaction analyses. Finally, we proposed that ROS could induce this immobilization mechanism by extracellular NET release mediated by TLR4. It could be an alternative mechanism by which granulocytes act to counter *A. phagocytophilum* dissemination in vertebrate hosts.

In conclusion, the results of the study support that A. *phagocytophilum* can manipulate host cells for its own benefit [15,25,26] through molecular mechanisms, including manipulation of the immune response and apoptosis in both tick vectors and vertebrate hosts [4,11,24,36,37]. In this process, the induction of NETosis by *A. phagocytophilum* constitutes a new mechanism triggered by pathogen infection to facilitate infection of human cells. 

The results of the study open the question of whether the induction of NETosis by *A. phagocytophilum* occurs also in tick vector cells. Future research should address this question and the possibility of interfering with *A. phagocytophilum*-induced NETosis to control pathogen infection and transmission and thus the risks associated with human and animal granulocytic anaplasmosis. 

## Data Availability

Data are included within the article.

## Supplementary Materials

The following supporting information can be downloaded at: https://www.mdpi.com/article/10.3390/vaccines10101756/s1, Figure S1: Showing the differentiation of human HL60 into neutrophils; Figure S2: Showing DNA levels of *A. phagocytophilum* in infected HL60 cells; Figure S3:A representative MPO levels measurement in the four treatments (X-VIVO control, *E. coli*, PMA, and *A. phagocytophilum*); Dataset S1: showing previously published proteomics data.
