# Supplementary material for: Low NETosis Induced in Anaplasma phagocytophilum-Infected Cells
Source: Vaccines (Basel). 2022 Oct 20;10(10):1756. doi: 10.3390/vaccines10101756 (PMC9610684; doi:10.3390/vaccines10101756)
Supplement: Supplementary file 1 [file vaccines-10-01756-s001.zip › Supplementary Materials.pdf]

Supplementary Material

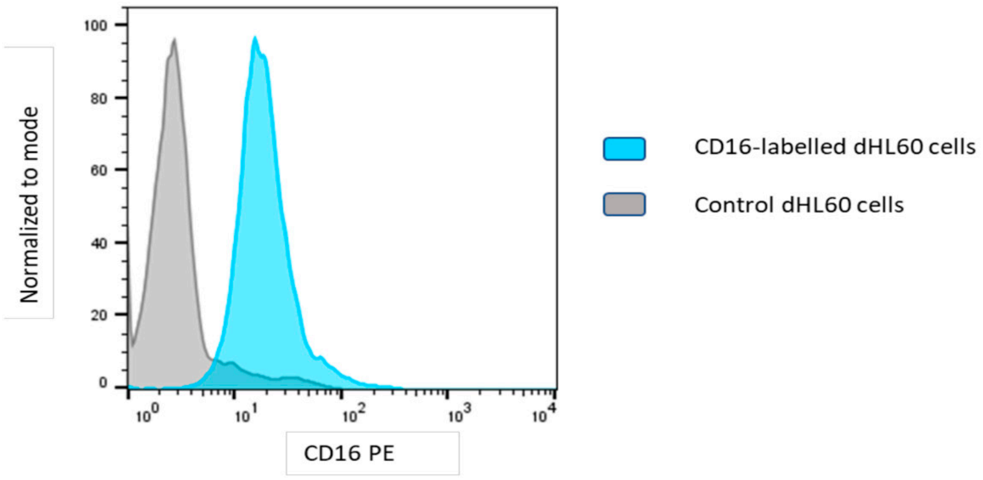

Figure S1. Differentiation evaluation of human HL60 into neutrophils. Differentiation was assessed by flow cytometry evaluating the expression of CD16 in DMSO-induced HL60 cells as a neutrophil surface marker after five days of induction. Two cell populations can be clearly distinguished between CD16-labelled differentiated cells (blue) and unlabelled DMSO-induced cells (grey).

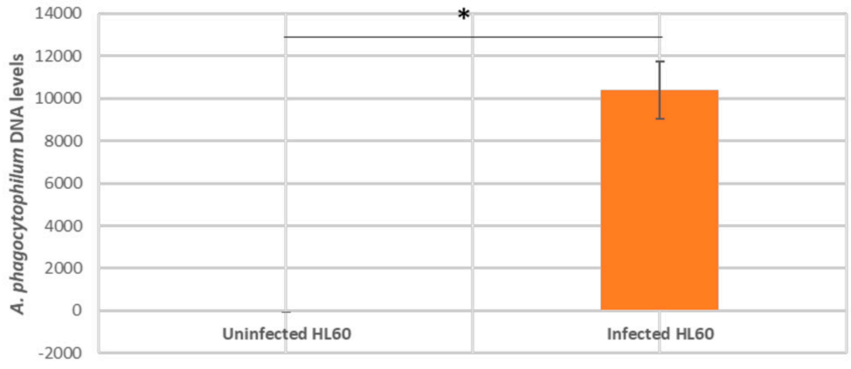

Figure S2. Evaluation of *A. phagocytophilum* levels in infected HL60 cells. The *A. phagocytophilum* DNA levels were determined by qPCR in infected HL60 cells. Normalized against human IPO8 gene Ct values (average+ S.D.) were compared between uninfected and infected cells by t-student (*$p<0.05$; n=3 biological replicates)

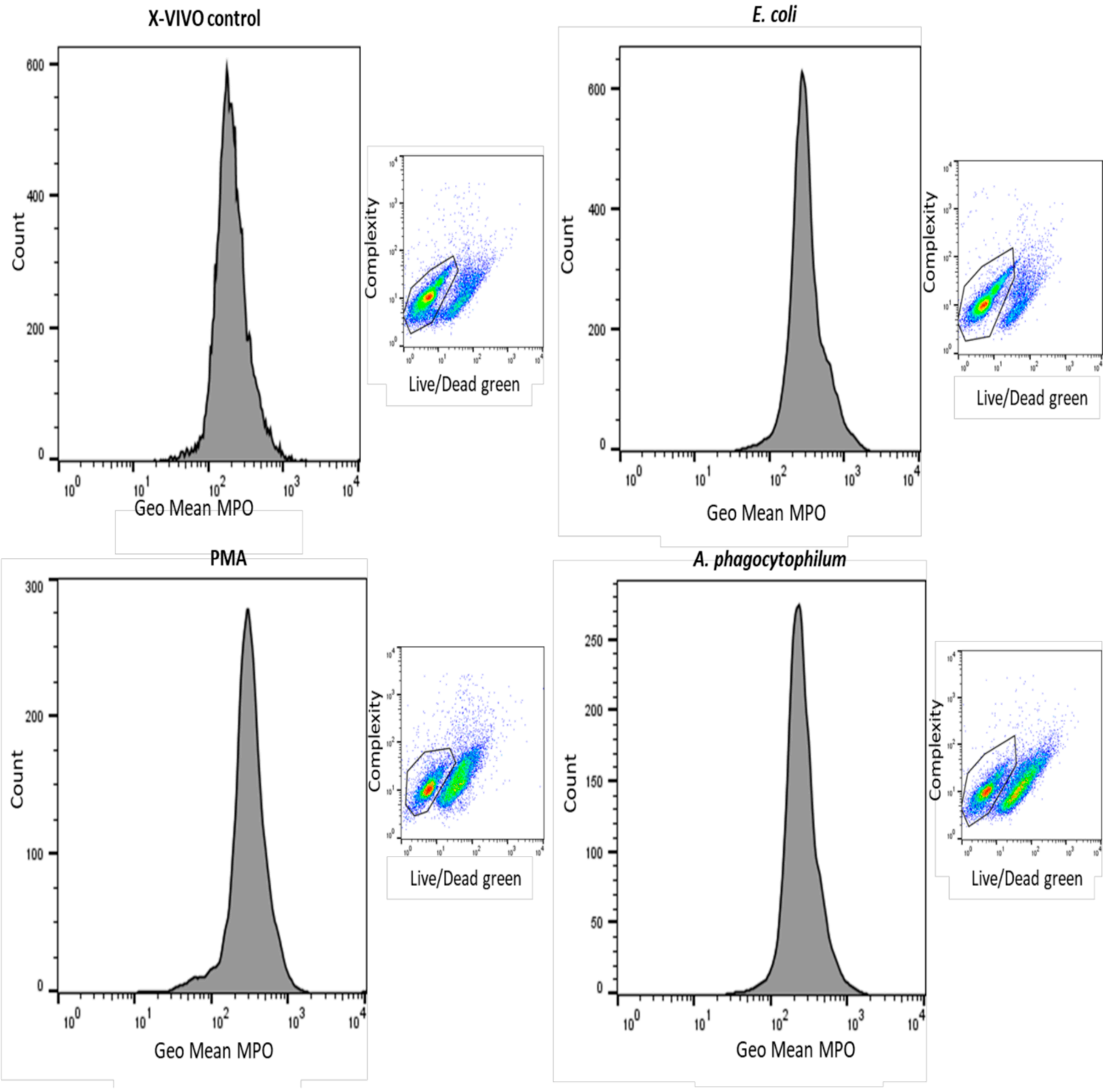

Figure S3. MPO expression levels were measured by flow cytometry. Representative MPO levels measured in the four treatments (X-VIVO control, *E. coli*, PMA and *A. phagocytophilum*) only in the gated live dHL60 cells (right graph) by Live/dead Fixable Green Dead cell stain Kit.
